# Macrolide-Resistant *Mycoplasma pneumoniae* Infections in Pediatric Community-Acquired Pneumonia 

**DOI:** 10.3201/eid2607.200017

**Published:** 2020-07

**Authors:** Yu-Chin Chen, Wei-Yun Hsu, Tu-Hsuan Chang

**Affiliations:** Chi-Mei Medical Center, Chiali, Tainan, Taiwan (Y.-C. Chen);; Chi-Mei Medical Center, Tainan, Taiwan (W.-Y. Hsu, T.-H. Chang)

**Keywords:** *Mycoplasma pneumoniae*, macrolide resistance, children, pneumonia, bacteria, Asia, antimicrobial resistance

## Abstract

A high prevalence rate of macrolide-resistant *Mycoplasma pneumoniae* (MRMP) has been reported in Asia. We performed a systematic review and meta-analysis to investigate the effect of macrolide resistance on the manifestations and clinical judgment during *M. pneumoniae* infections. We found no difference in clinical severity between MRMP and macrolide-sensitive *Mycoplasma pneumoniae* (MSMP) infections. However, in the pooled data, patients infected with MRMP had a longer febrile period (1.71 days), length of hospital stay (1.61 day), antibiotic drug courses (2.93 days), and defervescence time after macrolide treatment (2.04 days) compared with patients infected with MSMP. The risk of fever lasting for >48 hours after macrolide treatment was also significantly increased (OR 21.24), and an increased proportion of patients was changed to second-line treatment (OR 4.42). Our findings indicate diagnostic and therapeutic challenges after the emergence of MRMP. More precise diagnostic tools and clearly defined treatment should be appraised in the future.

*Mycoplasma pneumoniae* is a common causative pathogen in community-acquired pneumonia (CAP) during childhood. In the post–pneumococcal conjugate vaccine (PCV) 13 era, the epidemiology of pediatric pneumonia has changed. In some countries where PCV13 is already included in national immunization program, *M. pneumoniae* has become the leading pathogen in pediatric CAP (*1*,*2*).

The clinical manifestations of *M. pneumoniae* infection are usually mild and self-limited. However, life-threatening pneumonia or even acute respiratory distress syndrome requiring extracorporeal membrane oxygen has been reported (*3*). Furthermore, some extrapulmonary symptoms, such as mucositis, hepatitis, encephalitis, hemolysis, or erythema multiforme, have linked *M. pneumoniae* infection to the formation of autoimmunity or immune complexes. The association between *M. pneumoniae* and refractory asthma has also been mentioned (*4*).

Macrolides are the first-line therapy for *M. pneumoniae*. Because of high oral bioavailability and once-daily formulation, macrolides have been widely used in outpatient settings. During the past 10 years, however, macrolide-resistant *Mycoplasma pneumoniae* (MRMP) has emerged worldwide. The most prevalent area is Asia, where prevalence rates are 13.6%–100% (*5*). In Japan and China, resistance rates are >90% in some epidemic years (*5*,*6*).

The treatment of MRMP has become challenging. Although 1 report showed more complications in managing MRMP infections (*7*), the association between severe disease and resistance remains inconsistent and unclear. We conducted a systematic review and meta-analysis to examine the effect of macrolide resistance on the manifestations, outcomes, and clinical judgment of *M. pneumoniae* infection.

## Methods

### Search Strategy

We conducted a systematic literature search in PubMed, Embase, and the Cochrane Library database using the keywords *Mycoplasma pneumoniae*, macrolide, antibiotic resistance, and drug resistance. There was no language restriction in our search. We reviewed eligible full texts and the reference lists of the relevant studies. The last update of the study was on December 1, 2019.

Two independent reviewers (Y.-C.C. and T.-H.C.) screened all titles and abstracts for eligibility. Studies were eligible for inclusion if the study population was restricted to children (<18 years of age) with community-acquired pneumonia; macrolide resistance was detected by PCR including the 2 common point mutations, positions 2063 and 2064; and a direct comparator was used in the same cohorts (macrolide-sensitive *M. pneumoniae* [MSMP] group). We excluded review articles, editorial comments, case reports, and posters but included correspondence or letters that fulfilled these criteria.

### Data Extraction and Quality Assessment

After full-text screening for eligibility and review, the 3 authors extracted data independently of one another. We resolved disagreements by consensus or review by another reviewer. We extracted the following variables from each study, if available: author, journal, year of publication, study design, study country, time period, detected point mutations, clinical symptoms, total febrile days, length of hospital stay, defervescence days after macrolide, antibiotic history, laboratory results, and chest radiographic findings. We also extracted pediatric data from studies with both children and adults, if available. We assessed the quality of nonrandomized studies included in the meta-analysis using the Newcastle-Ottawa Scale and excluded articles with poor quality (score 0–3).

### Data Analysis

We used Review Manager software version 5.3 (Cochrane Collaboration, https://training.cochrane.org) and Comprehensive Meta-Analysis version 3 (Biostat, https://www.meta-analysis.com) for the analysis and conducted meta-analysis when >3 studies with available data reported the same outcome. We calculated heterogeneity (*I*^2^) to examine statistical heterogeneity across the included studies. We considered *I*^2^ >50% and p<0.05 to indicate substantial heterogeneity. We used random effects models to calculate odds ratios for binary outcomes and mean differences for continuous outcomes. We used Egger precision weighted linear regression tests and funnel plots to test potential publication bias. If publication bias was present, we used the trim-and-fill method and calculated Rosenthal’s fail-safe *N* to evaluate the effect.

## Results

### Study Characteristics

**Figure 1:**
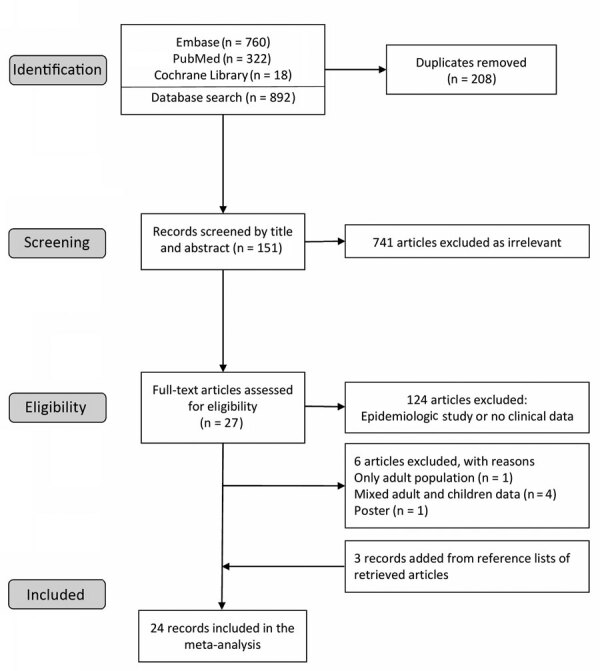
Flow diagram of selection process for meta-analysis of macrolide-resistant *Mycoplasma pneumoniae* infections in pediatric community-acquired pneumonia.

We identified 1,100 articles in the initial search (Figure 1). After removing duplicates, we screened 892 articles by titles and abstracts. We excluded obviously irrelevant articles and retrieved the remaining 151 for full text assessment. We then excluded epidemiologic or in vitro studies without clinical data. We included 27 full-text studies in the qualitative synthesis. We identified 3 records through manual search of the reference lists of retrieved articles. Finally, we included 24 full-text articles in the meta-analysis. The studies were conducted in the Asia-Pacific region, except for 1 in Italy. The range of resistance rates was 10%–88%. The A2063G transition mutation was detected in all studies (Table).

**Table:** Characteristics of the eligible studies of macrolide resistance and *Mycoplasma pneumoniae* infections.

Author	Study period	Country	Mutations detected	Disease entity	Case no.	Resistance
Chen (*8*)	2014–2016	China	A2063G, A2064G	CAP, inpatient	136	60%
Ma (*9*)	2010–2011	China	A2063G, A1290G	CAP, inpatient	57	63%
Xin (*10*)	2004–2005	China	A2063G, A2064G	CAP, inpatient	64	59%
Yuan (*11*)	2016	China	A2063G, A2064G, C2617G	CAP, inpatient	120	82%
Zhou (*7*)	2009–2010	China	A2063G, A2063T, A2064G	CAP, inpatient	235	88%
Cheong (*12*)	2011–2013	HK	A2063G	CAP, inpatient	93	27%
Lung (*13*)	2010–2013	HK	A2063G	CAP, inpatient	48	71%
Cardinale (*14*)	2010	Italy	A2063G, A2064G	CAP, inpatient	46	17%
Akashi (*15*)	2016–2017	Japan	A2063G, A2064G	CAP, mixed	222	65%
Ishiguro (*16*)	2013–2015	Japan	A2063G	CAP, mixed	109	54%
Kawai (*17*)	2005–2010	Japan	A2063G, A2064G	CAP, mixed	29	72%
Kawai (*18*)	2005–2012	Japan	A2063G, A2064G	CAP, mixed	188	80%
Matsubara (*19*)	2002–2006	Japan	A2063G, A2064G	CAP, NS	69	32%
Miyashita (*20*)	2008–2011	Japan	A2063G, A2064G	CAP, NS	71	59%
Okada (*21*)	2011	Japan	A2063G, A2064G	CAP, mixed	202	87%
Kim JH (*22*)	2011–2015	Korea	A2063G	CAP, inpatient	250	74%
Kim YJ (*23*)	2010–2015	Korea	A2063G	CAP, inpatient	107	10%
Lee (*24*)	2015	Korea	A2063G	CAP, mixed	94	13%
Seo (*25*)	2011	Korea	A2063G	CAP, inpatient	95	52%
Yoo (*26*)	2011	Korea	A2063G	CAP, mixed	91	30%
Yoon (*27*)	2010–2015	Korea	A2063G	CAP, inpatient	116	71%
Wu HM (*28*)	2011	Taiwan	A2063G	CAP, inpatient	73	12%
Wu PS (*29*)	2010–2011	Taiwan	A2063G	CAP, inpatient	60	23%
Yang (*30*)	2010–2017	Taiwan	A2063G, A2063T, A2064G	CAP, mixed	471	24%

*CAP, community acquired pneumonia; HK, Hong Kong; NS, not specified.

### Effect on Clinical Features and Outcomes

**Figure 2:**
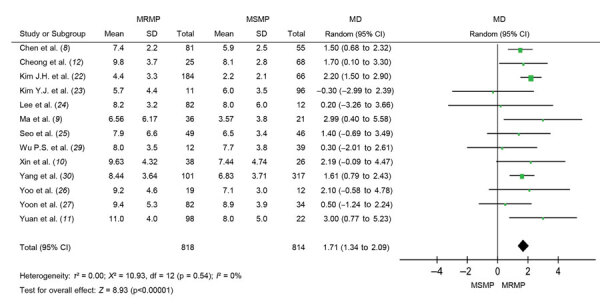
Forest plots of difference in total febrile days between MRMP and MSMP in meta-analysis of MRMP infections in pediatric community-acquired pneumonia. MD, mean difference; MRMP, macrolide-resistant *Mycoplasma pneumoniae*; MSMP, macrolide-sensitive *Mycoplasma pneumoniae*.

The duration of fever was longer in patients with MRMP than in patients with MSMP (mean difference [MD] 1.71, 95% CI 1.34–2.09; p<0.001) (Figure 2). The result was stable and consistent within studies (*I*^2^ = 0%; p = 0.54). MRMP infections were also associated with prolonged hospitalization compared with MSMP infections (MD 1.61, 95% CI 1.08–2.13; p<0.001) (Appendix Figure 1). We found no significant heterogeneity in the studies included (*I*^2^ = 28%; p = 0.18).

We examined the effect of macrolide resistance on work of breath and extrapulmonary symptoms. We found a slight trend toward MRMP patients with more extensive disease (Appendix Figure 2). Nevertheless, we found no difference in clinical features, such as dyspnea (OR 1.71, 95% CI 0.69–4.24; p = 0.24) or extrapulmonary manifestations (OR 1.31, 95% CI 0.85–2.02; p = 0.22), in patients with MRMP infections.

### Laboratory Results

We assessed inflammatory markers commonly examined during *M. pneumoniae* infection (Appendix Figure 3). Eleven studies provided data on leukocyte count; we found no significant difference between MRMP and MSMP patients (MD 0.09, 95% CI −0.31 to 0.50; p = 0.65). Nine studies assessed C-reactive protein (mg/L) during infection; again, we found no significant differences between MRMP and MSMP patients (MD −2.79, 95% CI −8.33 to 2.76; p = 0.32).

**Figure 3:**
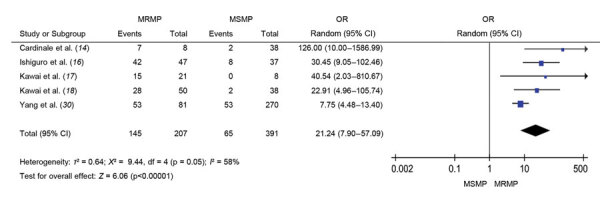
Forest plots comparing the pooled odds ratio of fever lasting for 48 hours after macrolide treatment between MRMP and MSMP in meta-analysis of MRMP infections in pediatric community-acquired pneumonia. MRMP, macrolide-resistant *Mycoplasma pneumoniae*; MSMP, macrolide-sensitive *Mycoplasma pneumoniae*; OR, odds ratio.

### Chest Radiographic Findings

We assessed the difference in chest radiographic findings in MRMP and MSMP patients (Appendix Figure 4). Neither consolidation ratio (OR 1.06, 95% CI 0.88–1.27; p = 0.52) nor pleural effusion (OR 1.19, 95% CI 0.70–2.03; p = 0.51) was influenced by macrolide resistance.

**Figure 4:**
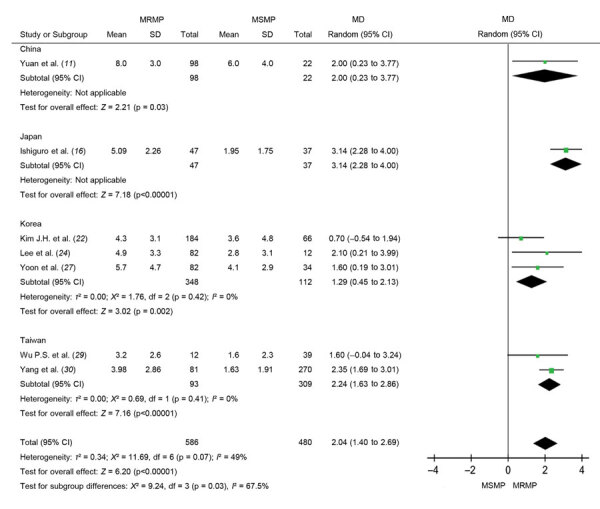
Forest plots depicting the defervescence time (days) after macrolide treatment in meta-analysis of MRMP infections in pediatric community-acquired pneumonia. Subgroup analysis was performed according to country. MD, mean difference; MRMP, macrolide-resistant *Mycoplasma pneumoniae*; MSMP, macrolide-sensitive *Mycoplasma pneumoniae*.

### Effect on Macrolide Efficacy and Antibiotic Prescription

In children with MRMP infection, fever may persist for >48 hours despite macrolide use. Figure 3 illustrates significantly increased odds of poor response to macrolide in patients with MRMP infections (OR 21.24, 95% CI 7.90–57.09; p<0.001).

Because efficacy of macrolides was reduced in patients with MRMP infections, we further investigated the exact effect of macrolide resistance on defervescence. The pooled results show significantly longer febrile duration (days) after ineffective treatment (MD 2.04, 95% CI 1.40–2.69; p<0.001). However, we observed an overall low-to-moderate heterogeneity within studies (*I*^2^ = 49%; p = 0.07). Considering different treatment policies (timing to initiate second-line antibiotic or corticosteroid) for *M. pneumoniae* among regions, we performed a subgroup analysis according to country (Figure 4). 

**Figure 5:**
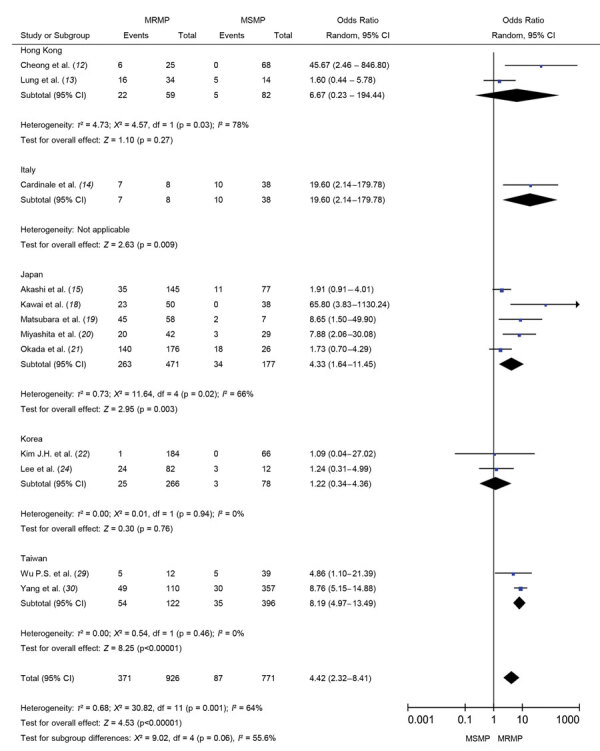
Forest plots comparing MRMP and MSMP by the pooled odds ratio of changing antibiotics in meta-analysis of MRMP infections in pediatric community-acquired pneumonia. MRMP, macrolide-resistant *Mycoplasma pneumoniae*; MSMP, macrolide-sensitive *Mycoplasma pneumoniae*; OR, odds ratio.

During macrolide treatment, some patients with *M. pneumoniae* infection would be switched to other classes of antibiotic drugs, such as the most commonly used fluoroquinolones and tetracyclines, that have different mechanisms of action (Figure 5). Increased proportions of patients were changed to second-line treatment when infected with MRMP (OR 4.42, 95% CI 2.32–8.41; p<0.001). Subgroup analysis divided by countries reveals that the heterogeneities were still high in Japan and Hong Kong.

**Figure 6:**
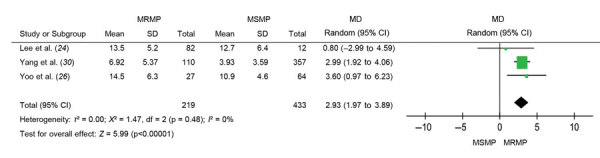
The duration difference (days) of antibiotic use between MRMP and MSMP infections in meta-analysis of MRMP infections in pediatric community-acquired pneumonia. MD, mean difference; MRMP, macrolide-resistant *Mycoplasma pneumoniae*; MSMP, macrolide-sensitive *Mycoplasma pneumoniae*.

The total duration of antibiotic drug treatment was longer when used to treat MRMP infections than when used to treat MSMP infections (MD 2.93, 95% CI 1.97–3.89; p<0.001) (Figure 6). There was no substantial heterogeneity (*I*^2^ = 0%; p = 0.48).

## Discussion

This systematic review and meta-analysis summarized currently available studies to compare the difference between MRMP and MSMP infections. The resistance rates varied within the studies, even in the same country (*5*). The overall resistance rate in large cohort studies in South Korea and Taiwan (*30*,*31*) increased over time; in contrast, the rate has gradually decreased in Japan since 2012 (*32*,*33*).

We applied multiple molecular methods to explain the spread of MRMP in Asia. Some reports using multilocus variable-number tandem-repeat analysis as the molecular typing method showed that the spread of *M. pneumoniae* seemed to be polyclonal (*34*,*35*). However, 2 recently published reports in South Korea and Japan that used multilocus sequence typing as the diagnostic method revealed that the wide spread of MRMP was associated with clonal expansion of the resistant ST3 clone (*31*,*36*). Whole-genome sequencing might be a better and more comprehensive tool for solving inconsistency and investigating *M. pneumoniae* evolutionary trends in the future.

The clinical manifestations, chest radiographic findings, and laboratory data were not altered by macrolide resistance. Although some studies showed more severe radiological findings (*30*) and more complications (*7*) after MRMP infections, there appeared to be no significant difference in the pooled data. *M. pneumoniae* presents a unique virulence factor in humans, an ADP-ribosyl transferase known as the community-acquired respiratory distress syndrome toxin (CARDS toxin). Lluch-Senar et al. (*37*) performed sequence analysis of the P1 adhesin gene and stated that type 2 strain produced more CARDS toxin. However, Zhao et al. (*38*) and Eshaghi et al. (*39*) failed to demonstrate this difference between MRMP and MSMP. Currently, no evidence supports the causal relationship between macrolide resistance and disease severity.

The efficacy of macrolide is significantly decreased during MRMP treatment compared to MSMP treatment. The most common point mutation in the domain V 23S rRNA is A2063G, which will cause great MIC increase to all macrolide drugs. Other than A2063G, some studies also reported A2064G mutation, which could result in decreased macrolide affinity and elevation of MIC (*21*). Based on these results, we expected to see much longer fever duration from ineffective treatment. However, the pooled data revealed only an interval difference of 1.71 days between fever durations in MRMP and MSMP infections. We further examined the exact days of patients being afebrile after macrolide treatment and the clinical judgment on antibiotic drug use. The study results showed significant heterogeneity. 

We then performed subgroup analysis by country. The results reflected different treatment policies among countries, even among institutions. Treatment selection for MRMP might modify the effect of macrolide resistance on clinical course. For instance, a report in South Korea (*22*) demonstrated less effect of resistance on macrolide efficacy. The possible reason is that steroids were given to 18.5% of patients with MRMP in this cohort, but not to MSMP patients (3%; p = 0.002). The initiation of corticosteroid treatment is early in South Korea (*40*) but reserved for refractory cases in Japan (*41*). Another study in China (*7*) noted that all patients in the report received only macrolides, given that the antimicrobial drug options are limited for preschool-age children. Therefore, more extrapulmonary complications (encephalitis, myocarditis, or hepatitis) occurred.

To treat or not to treat *M. pneumoniae* is still a dilemma to be resolved. MRMP treatment has raised another problem. Our meta-analysis identified 2 knowledge gaps. The first is the diagnostic gap. Macrolide resistance detection in most institutions relied on in-house PCR. Weighing the costs and benefits, it usually takes time to provide formal reports. Physicians usually base their suspicions of MRMP infections on clinical judgment of patients’ response to treatment. In Japan and Taiwan, if fever persists for 48–72 hours after macrolide treatment, second-line antimicrobial drugs, such as fluoroquinolone or tetracycline, would be considered (*42*,*43*). Delayed defervescence of 2 days after macrolide (Figure 4) could be explained by this clinical practice. Timeliness of diagnostic tests after disease onset can be a factor in confirming macrolide resistance. Real-time or point-of-care testing should be used to make the diagnosis more precisely and quickly.

The second challenge is the therapeutic gap. In Japan, the therapeutic efficacy of tosufloxacin and minocycline has been demonstrated in several studies (*16*,*18*,*21*). However, because of side effects and the development of new resistant strains, empirical treatment for MRMP, especially in endemic areas, is the subject of an ongoing debate. In addition, delayed effective antimicrobial treatment for *M. pneumoniae* has been found to be related to immune reaction, which may lead to prolonged or extrapulmonary disease (*30*). Macrolide resistance is one of the significant risk factors for delayed effective treatment. This finding partially explains why patients with MRMP infections showed a trend of more extrapulmonary manifestations or consolidation in 2 studies (*7*,*30*). Some randomized controlled trials indicated a positive effect on early corticosteroid treatment whether or not there was macrolide resistance (*44*,*45*). However, a retrospective study of a large database in Japan did not support this viewpoint (*46*). A well-designed randomized trial or meta-analysis should be considered to clarify the role of corticosteroids. In conclusion, the management of *M. pneumoniae* infection might need to be reappraised.

In addition to prolonged clinical courses, our study indicates the effect of macrolide resistance on antibiotic drug consumption and imprecision. Increased macrolide usage in primary healthcare settings, as well as unnecessary and inappropriate prescriptions to treat acute respiratory tract infections, are common in countries in Asia (*47**–**49*). Continuous selective pressure of routinely used antibiotic drugs and high population density can possibly explain the emergence of MRMP. The extent of *M. pneumoniae* simultaneously increased with rising resistance, further resulting in increased consumption of antibiotic drugs. Antibiotic stewardship should be promoted to reduce macrolide resistance.

Our meta-analysis has limitations. First, not all reported mutations (such as C2617G) were described or checked in the included studies. Because positions 2063 and 2064 accounted for most of the mutations and have been reported in all articles included in this analysis, this influence could be minimized. Second, co-infection was not excluded in all studies. The co-infection rate with *M. pneumoniae* is low in some studies (*1*,*12*,*30*). Nevertheless, how to discriminate between carriage and infection is still a key issue. A combination of PCR and serologic tests, such as measurement of *M. pneumoniae*–specific IgM-secreting cells, would be a better way to determine the role of macrolide resistance in the future (50). Third, the natural course of MRMP infection is modified because in institutions where physicians are alert to MRMP, second-line therapy or corticosteroids will be administered promptly. Although this bias existed in the initial selection process, it reflected the current clinical practice in MRMP-prevalent areas and the dilemma in management of MRMP.

In summary, our analysis found that MRMP infections are associated with longer febrile duration than MSMP infections. Decreased macrolide efficacy and increased ineffective antimicrobial drug use have also been found. The effect of macrolide resistance on disease severity is inconclusive, and there are still diagnostic and therapeutic gaps in the management of MRMP. Reappraisal of precise diagnostic tools and clearly defined treatment are needed.

Appendix Additional information about the study of macrolide-resistant pediatric *Mycoplasma pneumoniae* infections.
