# Supplementary material for: Macrolide-Resistant Mycoplasma pneumoniae Infections in Pediatric Community-Acquired Pneumonia
Source: Emerg Infect Dis. 2020 Jul;26(7):1382–91. doi: 10.3201/eid2607.200017 (PMC7323531; doi:10.3201/eid2607.200017)
Supplement: Appendix — Additional information about the study of macrolide-resistant pediatric Mycoplasma pneumoniae infections. [file 20-0017-Techapp-s1.pdf]

Article DOI: https://doi.org/10.3201/eid2607.200017

Macrolide-Resistant *Mycoplasma pneumoniae* Infections in Pediatric Community-Acquired Pneumonia

Appendix

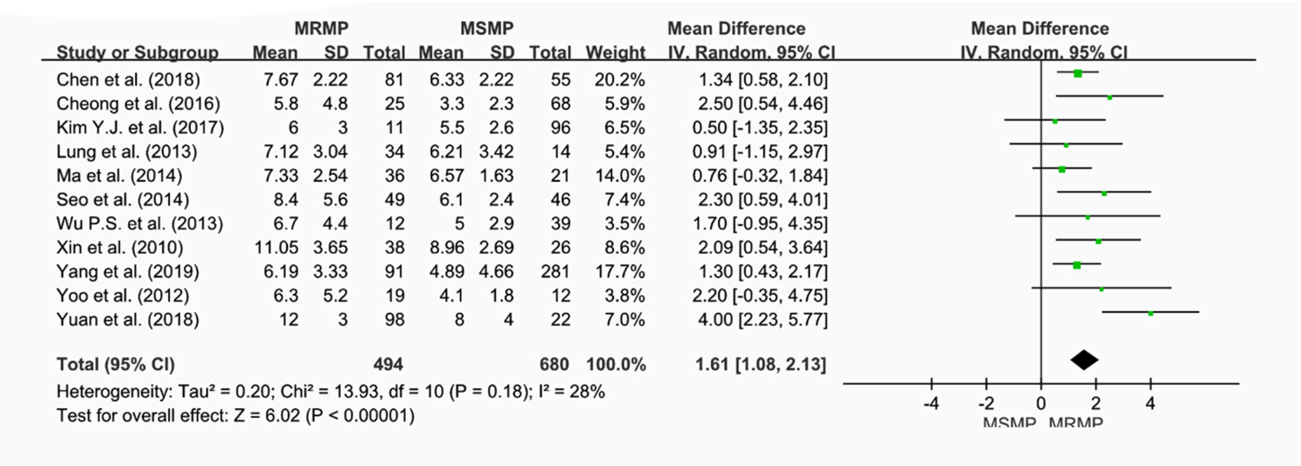

Study or Subgroup	MRMP Mean	MRMP SD	MRMP Total	MSMP Mean	MSMP SD	MSMP Total	Weight	Mean Difference IV, Random, 95% CI
Chen et al. (2018)	7.67	2.22	81	6.33	2.22	55	20.2%	1.34 [0.58, 2.10]
Cheong et al. (2016)	5.8	4.8	25	3.3	2.3	68	5.9%	2.50 [0.54, 4.46]
Kim Y.J. et al. (2017)	6	3	11	5.5	2.6	96	6.5%	0.50 [-1.35, 2.35]
Lung et al. (2013)	7.12	3.04	34	6.21	3.42	14	5.4%	0.91 [-1.15, 2.97]
Ma et al. (2014)	7.33	2.54	36	6.57	1.63	21	14.0%	0.76 [-0.32, 1.84]
Seo et al. (2014)	8.4	5.6	49	6.1	2.4	46	7.4%	2.30 [0.59, 4.01]
Wu P.S. et al. (2013)	6.7	4.4	12	5	2.9	39	3.5%	1.70 [-0.95, 4.35]
Xin et al. (2010)	11.05	3.65	38	8.96	2.69	26	8.6%	2.09 [0.54, 3.64]
Yang et al. (2019)	6.19	3.33	91	4.89	4.66	281	17.7%	1.30 [0.43, 2.17]
Yoo et al. (2012)	6.3	5.2	19	4.1	1.8	12	3.8%	2.20 [-0.35, 4.75]
Yuan et al. (2018)	12	3	98	8	4	22	7.0%	4.00 [2.23, 5.77]
**Total (95% CI)**			**494**			**680**	**100.0%**	**1.61 [1.08, 2.13]**

Heterogeneity: $Tau^2 = 0.20$; $Chi^2 = 13.93$, df = 10 (P = 0.18); $I^2 = 28\%$

Test for overall effect: Z = 6.02 (P < 0.00001)

Appendix Figure 1. Forest plots depicting the difference in length of hospital stay (days) between macrolide-resistant *Mycoplasma pneumoniae* (MRMP) and macrolide-sensitive *Mycoplasma pneumoniae* (MSMP).

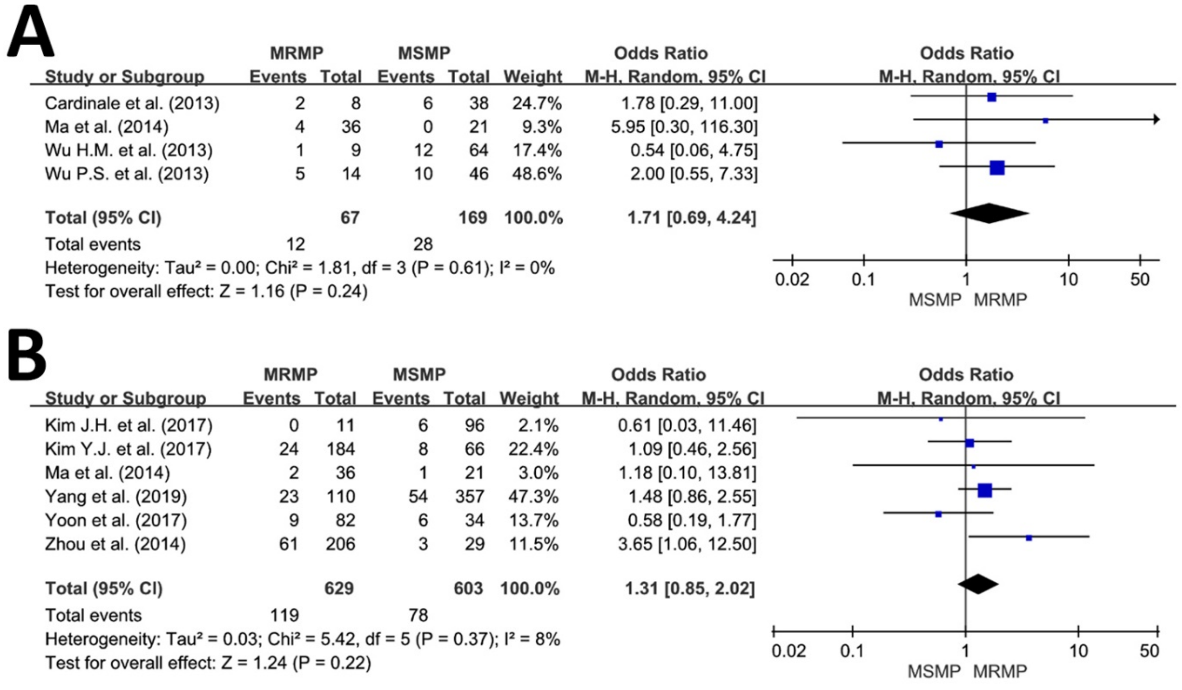

Appendix Figure 2. Forest plots comparing the clinical manifestations: A) dyspnea; B) extrapulmonary symptoms.

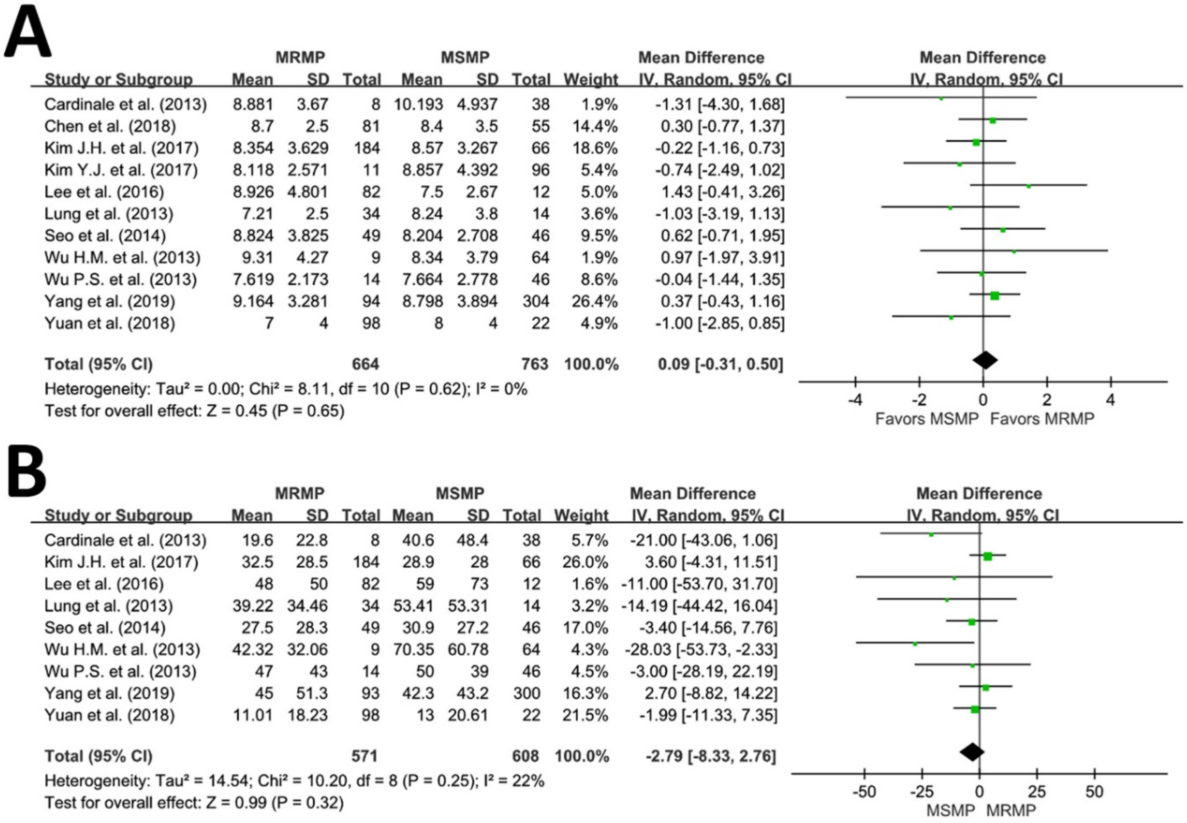

Appendix Figure 3. Forest plots of laboratory differences between macrolide-resistant *Mycoplasma pneumoniae* (MRMP) and macrolide-sensitive *Mycoplasma pneumoniae* (MSMP): A) leukocyte count ($\times 10^3$); B) C-reactive protein (mg/L).

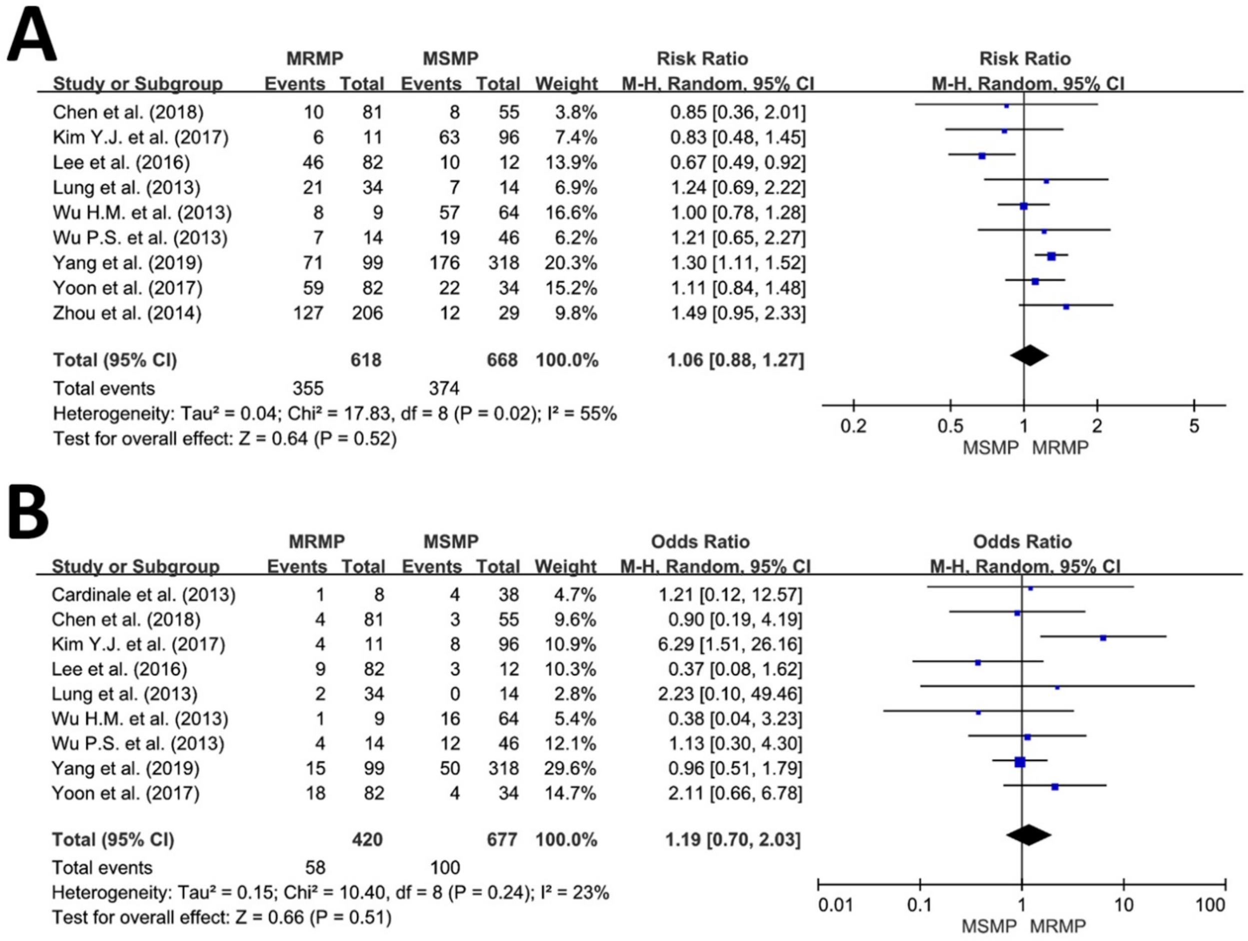

Appendix Figure 4. Forest plots illustrating the chest radiographic findings in *M. pneumoniae* infections:

A) consolidation; B) pleural effusion.
